# Reconsidering Ventriculoperitoneal Shunt Surgery and Postoperative Shunt Valve Pressure Adjustment: Our Approaches Learned From Past Challenges and Failures

**DOI:** 10.3389/fneur.2021.798488

**Published:** 2022-01-06

**Authors:** Shigeki Yamada, Masatsune Ishikawa, Madoka Nakajima, Kazuhiko Nozaki

**Affiliations:** ^1^Department of Neurosurgery, Shiga University of Medical Science, Shiga, Japan; ^2^Interfaculty Initiative in Information Studies/Institute of Industrial Science, The University of Tokyo, Tokyo, Japan; ^3^Department of Neurosurgery and Normal Pressure Hydrocephalus Center, Rakuwakai Otowa Hospital, Kyoto, Japan; ^4^Rakuwa Villa Ilios, Rakuwakai Healthcare System, Kyoto, Japan; ^5^Department of Neurosurgery, Juntendo University Faculty of Medicine, Tokyo, Japan

**Keywords:** idiopathic normal pressure hydrocephalus (iNPH), ventriculoperitoneal shunt (VP shunt), ventricles, cerebrospinal fluid (CSF), pressure adjustment and management, CSF tap test, DESH, preoperative simulation

## Abstract

Treatment for idiopathic normal pressure hydrocephalus (iNPH) continues to develop. Although ventriculoperitoneal shunt surgery has a long history and is one of the most established neurosurgeries, in the 1970s, the improvement rate of iNPH triad symptoms was poor and the risks related to shunt implantation were high. This led experts to question the surgical indication for iNPH and, over the next 20 years, cerebrospinal fluid (CSF) shunt surgery for iNPH fell out of favor and was rarely performed. However, the development of programmable-pressure shunt valve devices has reduced the major complications associated with the CSF drainage volume and appears to have increased shunt effectiveness. In addition, the development of support devices for the placement of ventricular catheters including preoperative virtual simulation and navigation systems has increased the certainty of ventriculoperitoneal shunt surgery. Secure shunt implantation is the most important prognostic indicator, but ensuring optimal initial valve pressure is also important. Since over-drainage is most likely to occur in the month after shunting, it is generally believed that a high initial setting of shunt valve pressure is the safest option. However, this does not always result in sufficient improvement of the symptoms in the early period after shunting. In fact, evidence suggests that setting the optimal valve pressure early after shunting may cause symptoms to improve earlier. This leads to improved quality of life and better long-term independent living expectations. However, in iNPH patients, the remaining symptoms may worsen again after several years, even when there is initial improvement due to setting the optimal valve pressure early after shunting. Because of the possibility of insufficient CSF drainage, the valve pressure should be reduced by one step (2–4 cmH_2_O) after 6 months to a year after shunting to maximize symptom improvement. After the valve pressure is reduced, a head CT scan is advised a month later.

## Introduction

Ventriculoperitoneal (VP) shunt surgery has a long history and is one of the most established neurosurgeries. The purpose of VP shunt surgery is to divert an accumulation of cerebrospinal fluid (CSF) from the cerebral ventricles to the abdominal cavity. The first VP shunt surgery was performed in 1905 by Kausch et al. but was unfortunately unsuccessful (1). The first clinically successful case used a shunt valve system to divert CSF from the ventricles to the jugular vein (2).

The Codman Company is a manufacturer of neurosurgical devices that began selling shunt valve devices for the treatment of hydrocephalus in the 1950s. Until the 1970s, however, several fixed differential pressure valves had been used in patients with idiopathic normal pressure hydrocephalus (iNPH), but the improvement rate of symptoms was poor and the risk of CSF over-drainage was extremely high. This led experts to question the surgical need for iNPH, and, for the next 20 years, CSF shunt surgery for iNPH was rarely performed. However, the Codman-Hakim Programmable Valve (CHPV) was developed in collaboration with Dr. Salomón Hakim, a Colombian neurosurgeon known to have named iNPH in the 1980s (3), and has been in use since 1992. Developments in shunt valve systems with programming pressure have dramatically reduced the problems of CSF over- and under-drainage and increased shunt effectiveness in elderly patients with iNPH (4–15). Nowadays, numerous patients with iNPH are treated with shunt surgery, according to the Japanese and international guidelines for the management of iNPH (16–21). This review was conducted to examine the history and current outcome of VP shunt surgery with recent shunt valve systems for iNPH patients. The latest knowledge of current surgical outcomes highlights the next issue to be addressed. The intention was to avoid repeating negative history of VP shunt surgery and to lead to the future of surgical intervention in elderly patients who require long-term care.

## Selection of Patients

### Evaluation of Symptoms

The best way to get better surgical outcomes is accurate diagnosis and careful selection of patients who are likely to improve with shunt surgery. The guidelines for the management of iNPH have consistently recommended to be used as some subjective evaluation scales along with objective and quantitative methods such as the 3-meter Timed Up & Go test (TUG) and Mini-Mental State Examination (MMSE) (18, 19, 21). As subjective evaluations, the grading scales for the severity of gait disturbance, cognitive impairment, and urinary incontinence and the modified Rankin scale have been used. In addition, as the pathological gait features specific to iNPH, small-step gait (short length of stride), broad-based gait (increasingly large step intervals), instability, difficulty in changing directions, frozen gait (no first step taken), and shuffle (dragging of feet and decreased elevation), are evaluated, respectively (21–24). However, it is known that different countries use different scales for these subjective assessments (25–29), and scale ratings do not match even among experts (23). Therefore, symptoms should be evaluated objectively using quantitative tests as much as possible. As reliable quantitative tests in patients with iNPH, TUG is widely used for assessment of gait and balance (14, 30, 31), and MMSE is for cognitive impairment (32). In addition, The European iNPH multicenter study adopted the Grooved Pegboard test and Stroop test as relatively simple neuropsychological tests (6, 29).

The CSF tap test is known as an invasive test useful for the diagnosis of iNPH before surgery (6, 13, 33–35), although it's also known to be a test with low sensitivity, that is, a high false negative rate. In a systematic review (36), the sensitivity was reported 58% (range: 26–87%) and the specificity was 75% (33–100%). Therefore, the CSF tap test is shifting its significance to a test for predicting the postoperative improvement of symptoms rather than for the diagnosis of iNPH (15, 37). There is no difference in the positive detection rate, sensitivity, and specificity with respect to the amount of CSF removed, in the range of 30 to 50 ml (38). Instead, the evaluation method and timing of assessing changes in clinical symptoms are more important (39). The time on TUG in the CSF tap test was proposed to be a reliable quantitative measure for predicting gait improvement after shunt surgery. The Japanese SINPHONI study showed that the patients whose TUG time shortened by ≥5 s at the CSF tap test had an almost 40% expectation of ≥10 s improvement on TUG time at 12 months after shunt surgery and an almost 65% expectation of ≥5 s improvement (14). In addition, the same study group recently showed that the iNPH patients whose MMSE improved by ≥3 points at the CSF tap test had an almost 50% expectation of ≥6 points improvement on MMSE at 12 months after shunt surgery, whereas the patients whose MMSE does not worsen at the CSF tap test had an almost 60% expectation of ≥3 points improvement on MMSE at 12 months after shunt surgery (32). However, the TUG time and MMSE were also found to be unsuitable for iNPH patients with mild symptoms.

To establish an internationally unified objective and quantitative evaluation of symptoms, a simple and accurate measurement method using modern smart devices owned by many people all over the world is suitable. By using an inertial gyroscope in a commercial smartphone, movements during the TUG can be automatically segmented into stand up, go forward, turn, go back, turn, and sit down (31). In addition, the chronological changes of acceleration of the trunk in three axial directions, forward-and-backward longitudinal acceleration, upward-and-downward vertical acceleration, and left-and-right horizontal acceleration can be precisely measured by using an accelerometer in a smartphone (40). For the iNPH patients with mild gait disturbance, the volume of 95% confidence ellipsoid for the tracks of the chronological changes of three dimensional (3D) acceleration during the TUG is a more important index for assessing gait severity than the time on TUG. Based on the results, we created the iTUG score, which simultaneously reflects both the iTUG time and the 3D acceleration volume. An iTUG score of 0 or less indicates inability to walk, and an iTUG score of 100 or more indicates healthy walking and no risk of falling (40). As an iPhone application that automatically calculates the iTUG score and automatically segments the six actions during TUG, Hacaro–iTUG (Digital Standard Co., Ltd., Osaka, Japan) can be freely downloaded from the Apple store (https://itunes.apple.com/us/app/hacaro-itug/id1367832791?l=ja&ls=1&mt=8). In addition, Hacaro–StroopTest (Digital Standard Co., Ltd.) is also available as a free download from the Apple Store (https://apps.apple.com/us/app/hacaro-strooptest/id1447081813) as a simplified version of the Stroop test that has been used in the European iNPH multicenter study. We recommend the iTUG score calculated by Hacaro–iTUG and the time measured by Hacaro–StroopTest as the next-generation international scales for assessing gait and cognitive dysfunction.

### Image Evaluation

The finding of ventricular enlargement on CT or MRI is essential for the diagnosis of iNPH. Although the etiology of ventricular enlargement in iNPH has not yet been elucidated, the total amount of intracranial CSF volume increases due to CSF malabsorption (41). The historically used Evans index >0.3 is defined as the maximum width of both frontal horns of the lateral ventricles divided by the maximum intracranial width on the same slice on CT or MRI. However, the lateral ventricles in iNPH are usually enlarged to the vertex direction rather than horizontal direction, because the Sylvian fissure is simultaneously enlarged by sandwiching the lateral ventricle from both sides. Therefore, the z-Evans index (42) which indicate the z-axial expansion of the bilateral ventricles is appropriate for evaluating ventricular enlargement and shunt effectiveness in iNPH, rather than the Evans index (43–48). By this simultaneous expansion of the ventricles and Sylvian fissure toward the top of the head, the high convexity and medial parts of the brain and subarachnoid spaces just above the lateral ventricles are compressed. This morphological characteristic of CSF distribution specific to iNPH has been called as Disproportionately Enlarged Subarachnoid-Space Hydrocephalus (DESH) (49, 50). As quantitative indicators of DESH, the callosal angle defined as the angle of the roof of the bilateral ventricles on the coronal plane at the posterior commissure (PC) level (51) and brain per ventricle ratios (BVRs) defined as the maximum width of the brain just above the lateral ventricles divided by the maximum width of the lateral ventricles on the coronal planes at the at the anterior commissure (AC) and PC levels (52) are useful not only for preoperative evaluation but also for comparison before and after shunt surgery (53, 54).

A state-of-the-art artificial intelligence technology is already able to automatically, accurately, and instantly segment the ventricles and subarachnoid spaces. Therefore, in the near future, it is expected that the diagnostic imaging of iNPH will become more accurate as the three-dimensional index of DESH such as convexity subarachnoid space per ventricle ratio (CVR) defined as the volume of the convexity subarachnoid space divided by the total ventricular volume (54) can be measured more easily.

### Surgical Indication

Only about half of the patients diagnosed with probable iNPH based on the image finding of DESH and response to the CSF tap test undergo shunt surgery (55, 56). Even if a patient is diagnosed with probable iNPH, the advantages (symptom improvement) and disadvantages (complications) of having shunt surgery should be evaluated individually for each patient. The major reasons why surgical indications are divided among neurosurgeons are patient's age and comorbid conditions such as Alzheimer's disease, stroke, and diabetes mellitus; heavy drinking, which increase the surgical risk and reduce improvement rates; and environmental factors including living alone without any caregivers or in domiciliary care or care homes. The majority of neurosurgeons may decide that the shunt surgery is not indicated, if a patient with probable iNPH has no decision-making ability and the cooperation of relatives cannot be obtained (57). The prediction of symptom improvement and surgical risks require consensus among the patients, neurosurgeons and referring physicians. Based on the evidence (10, 11, 49), neurosurgeons estimate that shunt surgery will improve only 20% of symptoms in 60–80% of iNPH patients, and a complete improvement of iNPH-related symptoms is very rare, even with the correct diagnosis of iNPH and perfect surgery and postoperative management (15). In addition, neurosurgeons are concerned about unexpected surgical complications and co-existence of comorbid degenerative diseases that worsen symptoms after shunting. Super-seniors aged 85 years old or over had the higher comorbidity rate of stroke and dementia, and these comorbidities are known to be the cause of diminished effectiveness of shunting and shortened sign improvement period. In addition, super-seniors diagnosed with iNPH often have a decreased walking speed, decreased physical activity, and muscle weakness, so then they already meet the criteria for frailty at the time of iNPH diagnosis, and are vulnerable to external stress. Super-seniors with frailty syndrome have a risk of being unable to maintain their preoperative living functions due to hospitalization or shunt surgery. In addition, the risk of shunt infection increases due to the deterioration of the skin's protective function and immunity due to aging, and if a shunt infection should occur, there will be a period during which the patient cannot stand or walk, and the symptoms will remain worse than before surgery at the discharge. In addition, because the super-seniors usually have their cerebral atrophy, the risk of chronic subdural hematoma is also increased due to CSF overdrainage and falls. Therefore, surgical intervention should be considered after fully explaining to the patient and their caregiver that the possibility of exacerbation is not unexpected.

## Surgical Techniques and Postoperative Clinical Management

### Lateral Ventricle Approach

**Figure 1:**
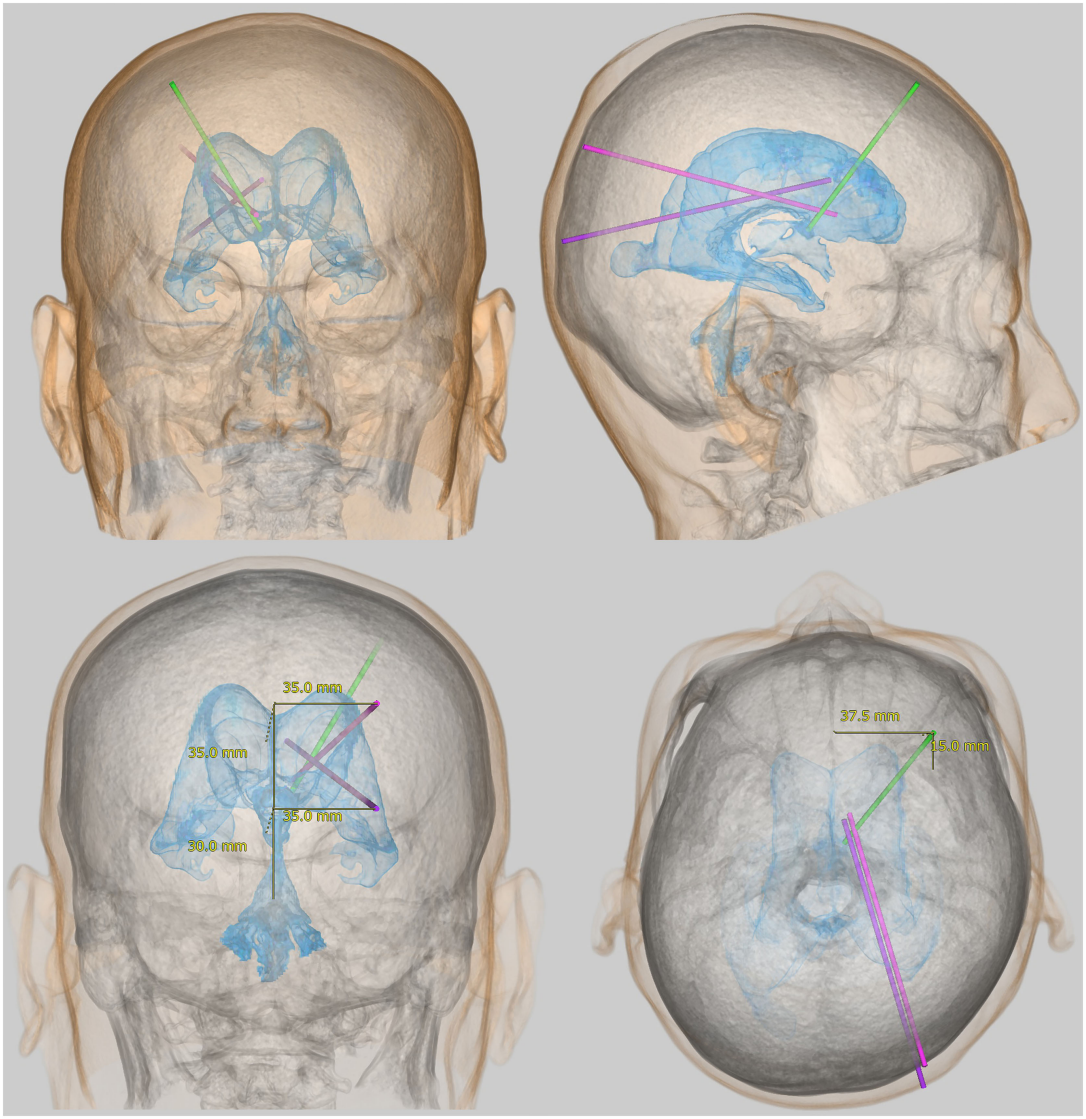
Representative approaches for placement of a ventricular catheter in the lateral ventricle. The light green bar indicates the root for the frontal approach from the coronal point, the purple bar indicates the root for the occipital approach from Dandy's point, and the pink bar indicates the root for the parietooccipital approach from Frazier's point.

The lateral ventricles are C-shaped cavities at the center of the cerebrum divided into the frontal (or anterior), occipital (or posterior), and temporal (or inferior) horns; the trigone (or atrium); and the body. In general, access to the lateral ventricles is by the frontal, occipital, or parietooccipital approach. The frontal approach is the most frequently used in this procedure. The coronal point, which is located 1–2 cm anterior to the coronal suture and 3–4 cm lateral from the midline, is the most common site for ventricular puncture (Figure 1), and the eyes, nose, and ears can be used as surface anatomical landmarks. However, the frontal approach in VP shunting requires the head to rotate because the silicone tube passes under the scalp behind the ear. This makes entry to the frontal horn of the ipsilateral lateral ventricle difficult, compared to the external ventricular drainage in which the head position is maintained in the median position (58). In addition, patients with iNPH sometimes exhibit smaller lateral ventricles, especially the frontal horn due to the enlargement of Sylvian fissure and basal cistern (42, 45, 49, 52, 59). The occipital and parietooccipital approaches are thought to make entry into the ipsilateral lateral ventricle more difficult. Using the occipital approach, the nearest entry point from the occipital horn of the lateral ventricle is Dandy's point, located 3 cm above and 2 cm lateral to the external occipital protuberance (inion). In the parietooccipital approach, Frazier's point is the most suitable entry point. This is located 6 cm above and 4 cm lateral to the inion. While Frazier's point is further from the ventricle, it provides easier catheter insertion into the body of the ipsilateral lateral ventricle than Dandy's point, as shown in Figure 1. However, ventricular puncture from Dandy's point in the occipital approach provides easier catheter insertion into the temporal horn of the lateral ventricle. With entry through both Dandy's point and Frazier's point, the ears and inion are the useful superficial landmarks. Therefore, while these points offer a cosmetic advantage but a higher risk of inappropriate catheter placement than the coronal point for the frontal approach. If the puncture point moves for any reason, there is the possibility of accidental puncture of the venous sinus. In particular, Dandy's point has a higher risk of bleeding than Frazier's point because it is close to both the superior sagittal sinus and the transverse sinus. However, using the preoperative simulation described below, the parietooccipital approach from Frazier's point can more accurately place the ventricular catheter in the optimal position than the frontal approach in VP shunt surgery.

### Preoperative Simulation for Ventricular Catheter Placement

Although VP shunt surgery is considered a relatively simple operation, few neurosurgeons are confident in their ability to position a ventricular catheter correctly with the first puncture. Misdirected brain puncture risks damaging functionally important locations such as the thalamus, basal ganglia, and corona radiata. In addition, inappropriate catheter placement, for example, with the ventricular catheter tip inserted into the brain, significantly increases the risk of shunt malfunction (58, 60–62). Therefore, it is necessary to pay special attention to the appropriate placement of the ventricular catheter in VP shunt surgery. Recent studies have found that the success rate of the frontal approach with the free-hand technique is only 44–64% (58, 60, 63–65), whereas that of the occipital or parietooccipital approach is 67–71% (62, 66). Because the possibility of inappropriate placement is significant in all free-hand approaches, ventricular catheter placement should not be performed by free-hand puncture. Some neurosurgeons prefer to use intraoperative imaging guidance instruments, such as ultrasonography, stereotaxy, and neuro-navigation. These have been shown to provide significantly greater accuracy than free-hand puncture (58, 62, 64, 65, 67, 68). An alternative to intraoperative imaging is preoperative simulations to determine the entry point, direction, and depth of puncture. With a frontal approach from the coronal point, it is not possible to determine the target on the scalp by using preoperative simulation, whereas a parietooccipital or occipital approach allows accurate determination of the scalp target. Thus, a posterior ventricular puncture using preoperative simulation has a higher success rate due to accurate determination of the entry and target points.

We have described as concretely as possible so that many neurosurgeons can utilize our method, preoperative 3D simulation of ventricular catheter placement using the parietooccipital approach from Frazier's point.

We usually perform the computational simulation twice using a 3D workstation (Synapse 3D; Fujifilm Medical Systems, Tokyo, Japan) before shunt surgery (Supplementary Video 1). The first simulation is to determine the optimal route and entry and target points, and the second is to confirm the measurement points where the markers have been placed.

**Figure 2:**
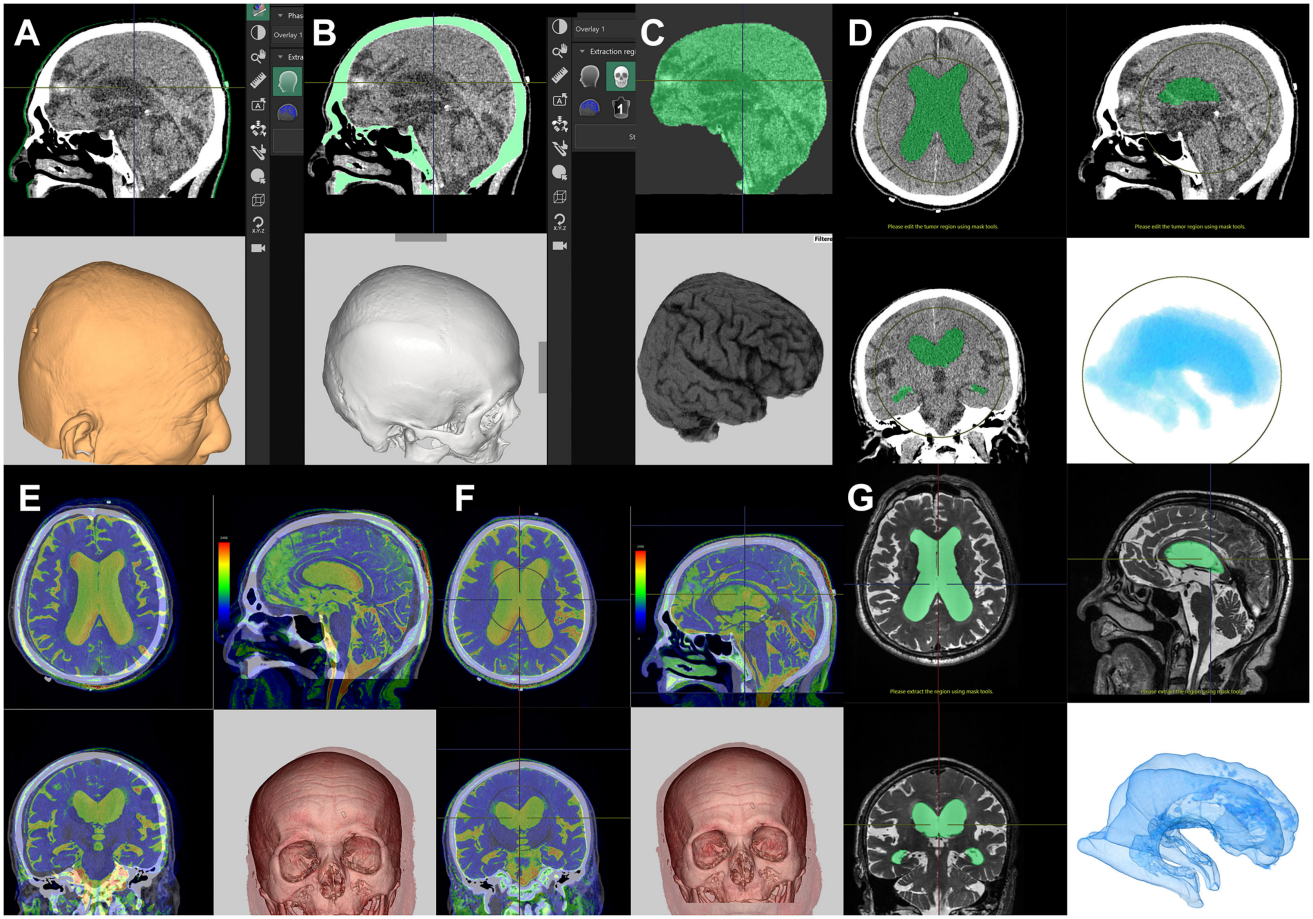
Preparation for computational virtual simulation of ventricular puncture. Screenshots of the Craniotomy/Tensor Analysis application on the SYNAPSE 3D workstation. Clicking the buttons causes the following three imaging components to be extracted automatically from the plain head CT scan: scalp **(A)**, skull **(B)**, and brain **(C)**. After the automated extraction, the lateral ventricle images were manually extracted from the brain **(D)**. If the patient has had a 3D-MRI for iNPH diagnosis, the T2-weighted 3D MRI is superimposed on the CT scan **(E)**. The CT and superimposed MRI images are automatically aligned with a single click **(F)**. The lateral ventricle images from T2-weighted 3D-MRI images **(G)** are easier and clearer to read than CT images **(D)**.

**Figure 3:**
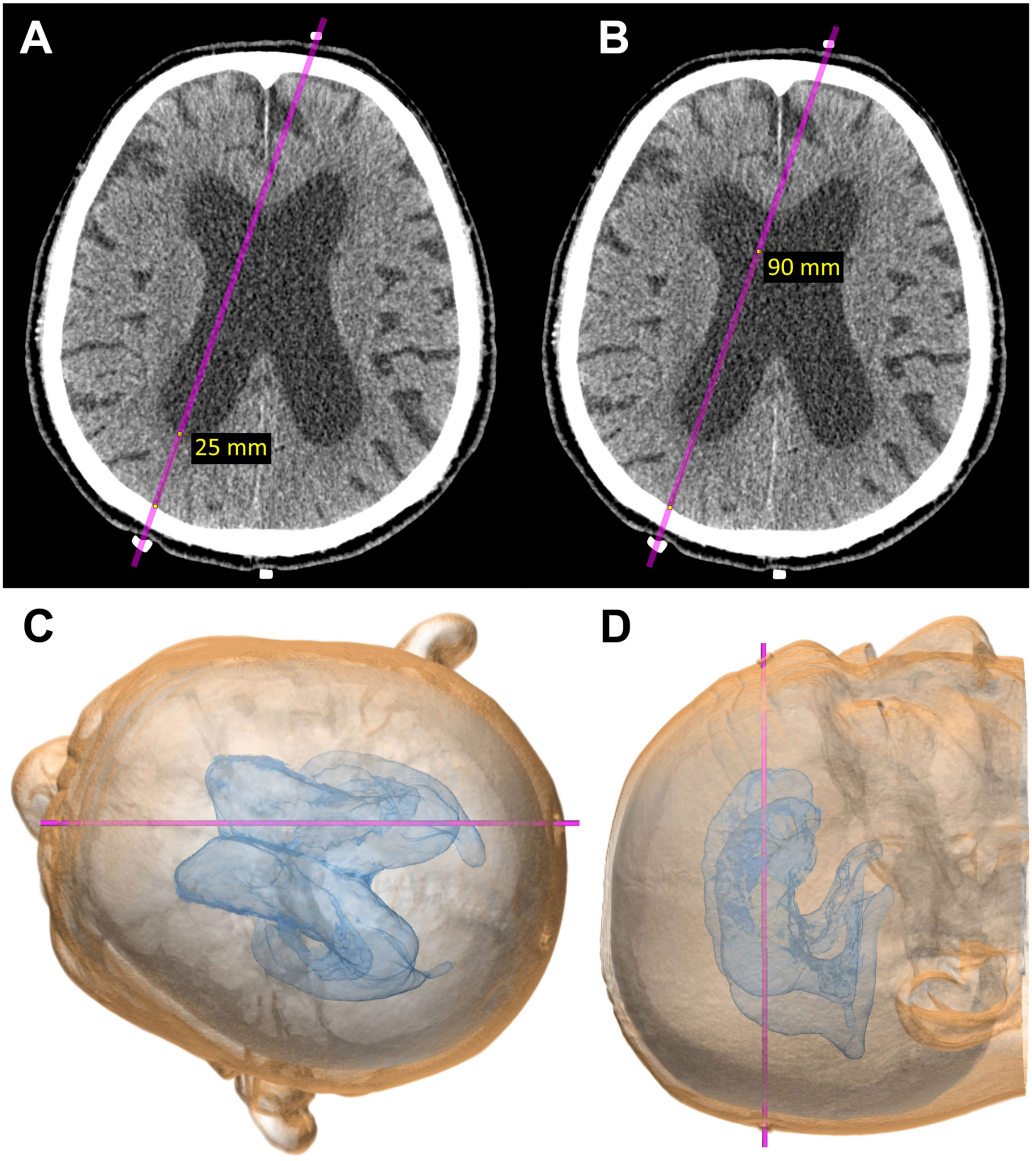
Computational virtual simulation of ventricular puncture in parietooccipital approach from Frazier's point. The depth from the brain surface to the entry point of the posterior horn of the lateral ventricle **(A)** and the length planned for ventricular catheter placement **(B)** were measured on the CT images. The virtual head position for surgery, setting the entry–target line parallel to the horizontal plane, was observed from the vertex angle **(C)**, and from above, perpendicular to the horizontal plane **(D)**.

Using the application tool Craniotomy/Tensor Analysis on the Synapse 3D workstation (https://healthcaresolutions-us.fujifilm.com/enterprise-imaging/synapse-3d), images of the scalp, skull, and brain can be automatically extracted from a plain helical CT scan within a few minutes (Figures 2A–C). A helical CT scan provides higher detectability of the scalp and skull than a 3D MRI. After automatic extraction of brain images, ventricle images can be obtained using techniques such as cutting, threshold extraction, and user-steered live-wire segmentation in the same application (Figure 2D). The extraction of images of CSF spaces using a simple threshold algorithm is preferential as these images can be superimposed on T2-weighted 3D MRI (Figures 2E–G), which provides clearer imaging of the boundary between the brain and the CSF than a CT scan. The annotation bar, with a diameter of 2 mm and a length of 180–200 mm, is useful for deciding the optimal direction for placement of the ventricular catheter (Figure 3). The priority in making this decision is to find the shortest pathway of brain penetration and the longest pathway in the ipsilateral ventricle. The entry and target points on the scalp are set to pass through the annotation bar on the scalp. The ideal target point in the brain is the genu of the corpus callosum and the anterior median wall of the bilateral ventricles. Therefore, the target point on the scalp is typically located 3–5 cm superior to the contralateral inner eye angle, using the nasion as a facial landmark.

A rubber eraser is the best marker for CT scans because it creates fewer artifacts and is easy to see even if it is small. To confirm the measured points for entry and target before shunt surgery, patients undergo a CT scan after setting three markers made using the tip of a rubber eraser, as shown in Figure 3. The first marker is placed at the planned entry point, the second at the planned target point on the scalp, and the third marker at the occipital midline, 6–8 cm superior to inion. This third marker is useful for the correction of the entry point.

During the second simulation to confirm the marker locations (Figure 3), the direction for ventricular catheter placement can be corrected if it is not ideal. It is preferable to correct the frontal marker rather than the occipital marker because the frontal marker has more facial landmarks, including the eyes and nose that help to guide the direction and find the midline. However, the third marker at the occipital midline is useful for the correction of the entry point. After determination of the entry point and the optimal direction for ventricular catheter placement, the depth from the brain surface to the entry point of the posterior horn of the lateral ventricle (Figure 3A) and the length planned for ventricular catheter placement (Figure 3B) are measured. In the final part of the preoperative simulation, the patient's head is virtually rotated to the surgical position, with the entry–target line parallel to the horizontal plane (Figure 3C) and observed from a top view perpendicular to the horizontal plane (Figure 3D).

### Shunt Valve Selection

All pressure-controlled shunt valves are manufactured to operate within a specific pressure range. As the history of iNPH treatments has shown, CSF shunt surgery using fixed-pressure valves for iNPH produces poor prognoses. However, some previous studies have found that fixed-pressure valves are still used more frequently than programmable-pressure shunt valves (5, 69, 70). Elderly patients who use the medium pressure valve have a high shunt revision rate due to issues with over-drainage (subdural hematoma) or under-drainage (less effective than expected). Brain plasticity in elderly patients with iNPH seems to be lower than that seen in young patients with secondary NPH. Since the therapeutic effects of iNPH have been recognized since the development and distribution of programmable-pressure shunt valves, it seems obvious that this type of shunt valve should be used for the treatment of iNPH. It is possible that the preference for fixed-pressure valves is due to financial constraints, but when the costs of re-operation are taken into account, it is cheaper to use a programmable-pressure shunt valve (12, 71). Programmable-pressure valves allow the neurosurgeon to choose a pressure setting before surgery and easily change them afterward based on the needs of the patient. Until 2015, our iNPH patients used the CHPV (Integra LifeSciences Corp., USA), which has 18 operating pressure settings in 10 mmH_2_O increments from 30 to 200 mmH_2_O (3). In 2015, the Codman CERTAS Plus Programmable Valve (Integra LifeSciences Corp., USA) was developed. This is an MRI-resistant programmable valve with seven operating pressure levels from 36 to 238 mm H_2_O and “virtual off (8),” which sets the valve pressure consistently above 400 mmH_2_O (72). Considering the easy adjustment and confirmation of valve pressure, there is no need for re-adjustment after 3Tesla MRI, and the virtual off function offers greater advantages for iNPH patients than does the fine pressure setting in CHPV. Therefore, the CERTAS valve has been the first choice for our VP shunt surgery since 2015. In addition, to prevent over-drainage related to body posture, we routinely use the Siphonguard Anti-Siphon Device (Integra LifeSciences Corp., USA). This is an automatic hydrodynamic switch that increases resistance to reduce the CSF drainage rate below 0.4 ml/min when it rises above 0.6 ml/min (73).

### Surgical Procedure

**Figure 4:**
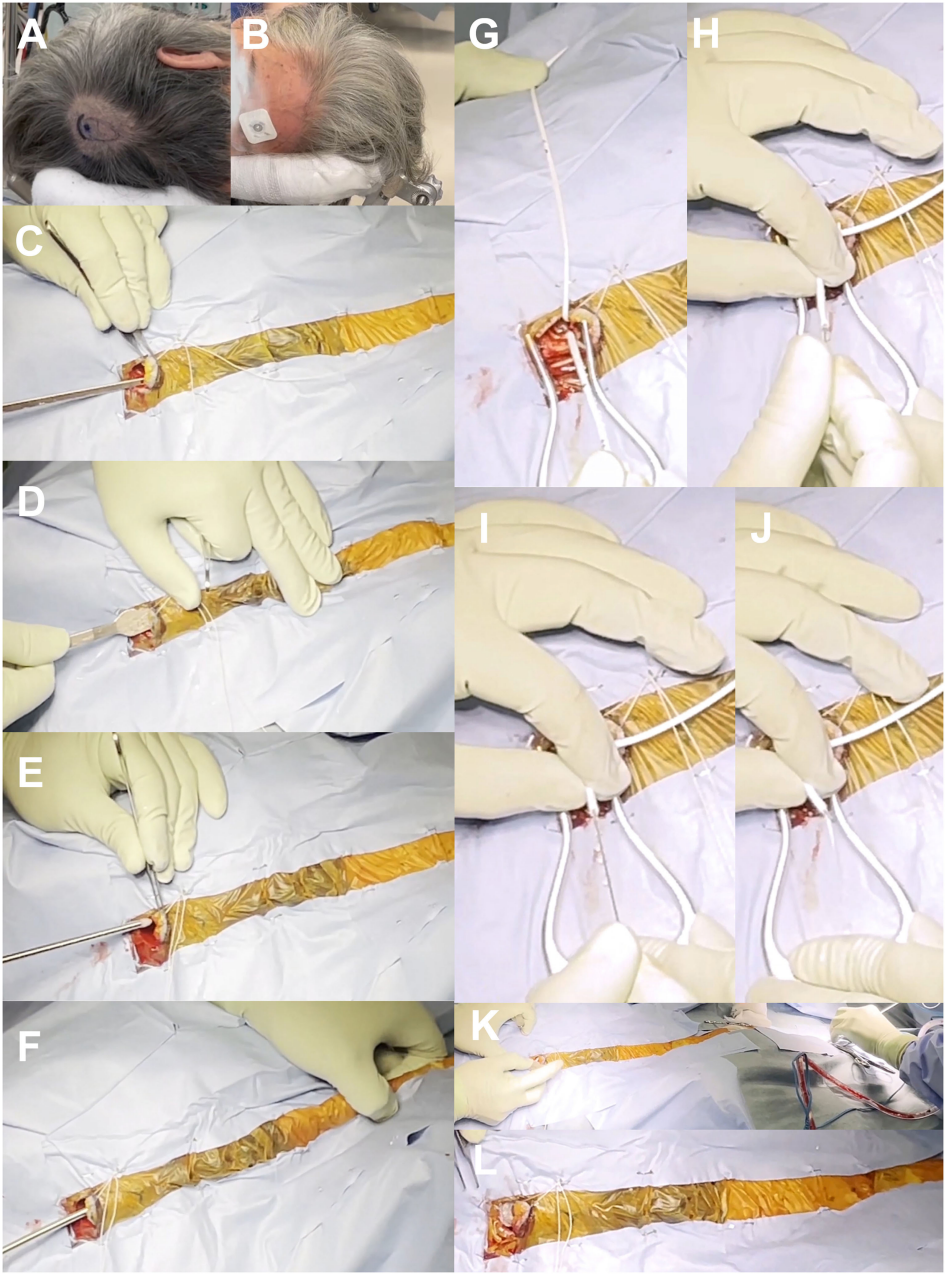
Surgical photographs. Marking of skin incision and valve placement position **(A)**, putting electrocardiogram sticker on the target point of scalp **(B)**, subcutaneous tunnel using spatula-shaped passer **(C,D)** and long cylindrical passer **(E,F)**, insertion of ventricular catheter **(G–J)**, and placement of shunt valve **(K,L)**.

**Figure 5:**
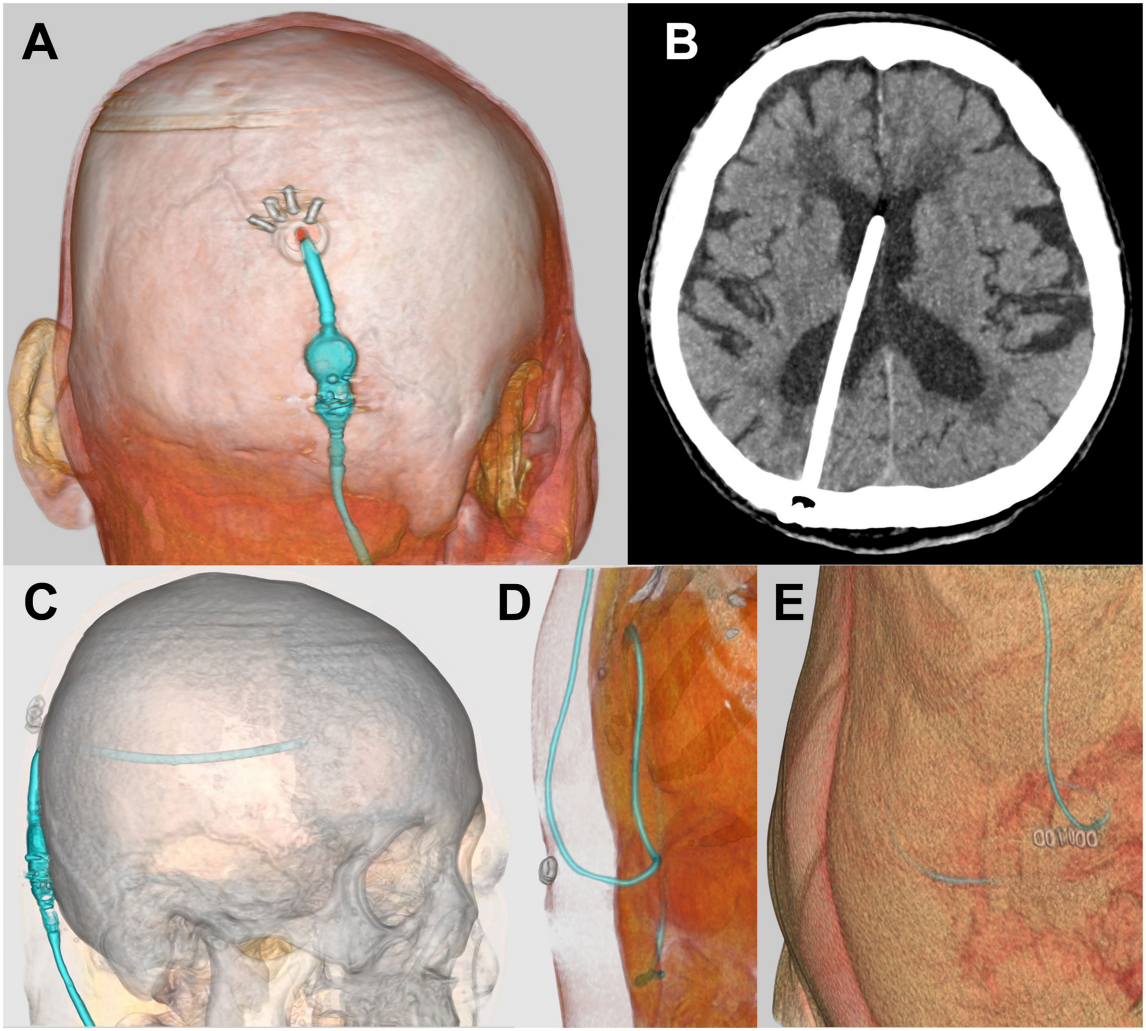
Postoperative CT images. Postoperative head CT images **(A–C)** and abdominal CT images **(D,E)** created by SYNAPSE 3D workstation were confirmed.

As described above, the first choice for our VP shunt surgery is a right-side parietooccipital approach (Figure 4A). Patients are placed with a pillow under the right shoulder and a slightly arched back to ensure shallow abdominal depth. The patient's head is carefully placed so that the entry–target line is horizontal to the ground in the same way as in the preoperative 3D simulation. An electrocardiogram sticker is placed on the left side of the forehead at the measured scalp target (Figure 4B). The right parietooccipital scalp is incised at the entry point, at ~2 cm on the slightly curved protrusion toward the vertex, and the periosteum is retracted (Supplementary Video 2). A skin incision is then made ~2 cm above the right rectus abdominis muscle. The subcutaneous tunnel is passed from the cranial side to the abdominal side using two types of passers (Supplementary Video 3). The first short spatula-shaped passer is placed subcutaneously from the scalp incision to just above the collarbone (Figures 4C,D). This is then changed to a long cylindrical passer to the abdominal incision without a relay incision to prevent infection (Figures 4E,F). During tunneling, the passer is carefully positioned to prevent deep insertion, particularly under the collarbone, checking the tip position visually and by touch. A small hole is drilled in the skull, and a cross is incised on the dura. A ventricular catheter with an inlet stylet is then directly punctured without a trial puncture using a metal puncture needle (Supplementary Video 3). A ventricular catheter is inserted horizontally to the ground while considering the direction toward the target from the top viewpoint (Figure 4G). When the ventricular catheter is inserted into the posterior horn of the lateral ventricle, a small amount of CSF leaks through the side of the inlet stylet. After confirming the clear CSF droplet, the ventricular catheter is slowly advanced to the simulated length, and the inlet stylet is removed (Figures 4H–J). The catheter is placed in the anterior horn or body of the ipsilateral ventricle to avoid choroid plexus involvement. After confirming smooth CSF drainage, the ventricular catheter is cut to an adequate length and connected with the shunt valve system. Finally, the patency of the shunt valve system is confirmed by pushing the shunt reservoir (Figures 4K,L). After the operation, the position of the shunt valve system is confirmed by a CT scan (Figure 5).

### Initial Setting of Shunt Valve Pressure

Over-drainage is most likely to occur early after CSF shunt surgery, so it is safer to set the initial pressure higher than the estimated optimal pressure. However, this can prevent sufficient improvement of the patient's symptoms due to under-drainage. If the valve pressure is initially set low to drain sufficient CSF, the symptoms may improve early after the shunt surgery, but the risk of over-drainage is also increased.

Miyake et al. have produced a quick reference table that uses the patient's height and weight (body mass index) to estimate the optimal initial pressure setting (74). This quick reference table was adopted in a Japanese study of iNPH on neurological improvement (SINPHONI) in which 100 patients with iNPH underwent VP shunt surgery using the CHPV. They observed relatively good outcomes and a low complication rate, but they were unable to prove that the quick reference table is superior to other methods because this was a single-arm study (11, 49, 75).

Because NPH is defined as “normal pressure” chronic hydrocephalus, it is widely believed that valve pressure fixed at medium (normal range pressure of human CSF) is sufficient for iNPH. In a prospective, double-blind, randomized controlled study using a CHPV, iNPH patients were divided into two groups (9). The first group underwent a monthly reduction of the valve pressure in steps of 40 mmH_2_O from an initial pressure of 200 mmH_2_O to a final pressure of 40 mmH_2_O over 4 months. The second group had valve pressure set and maintained at 120 mmH_2_O. In the first group, the improvement rate 6 months after VP shunt surgery was 88%, while that in the second group was 62%. However, the complication rate due to over-drainage was significantly higher in the second group with a consistent valve setting of ≤120 mmH_2_O. A second report by the same authors found improvement rates in quantitative assessments of steps taken during a 10 m walk, Stroop tests, and Grooved Pegboard tests but with no significant differences between the two groups (76). Therefore, they recommended setting the initial shunt valve pressure at 120 mmH_2_O and not reducing it lower than 120 mmH_2_O.

**Figure 6:**
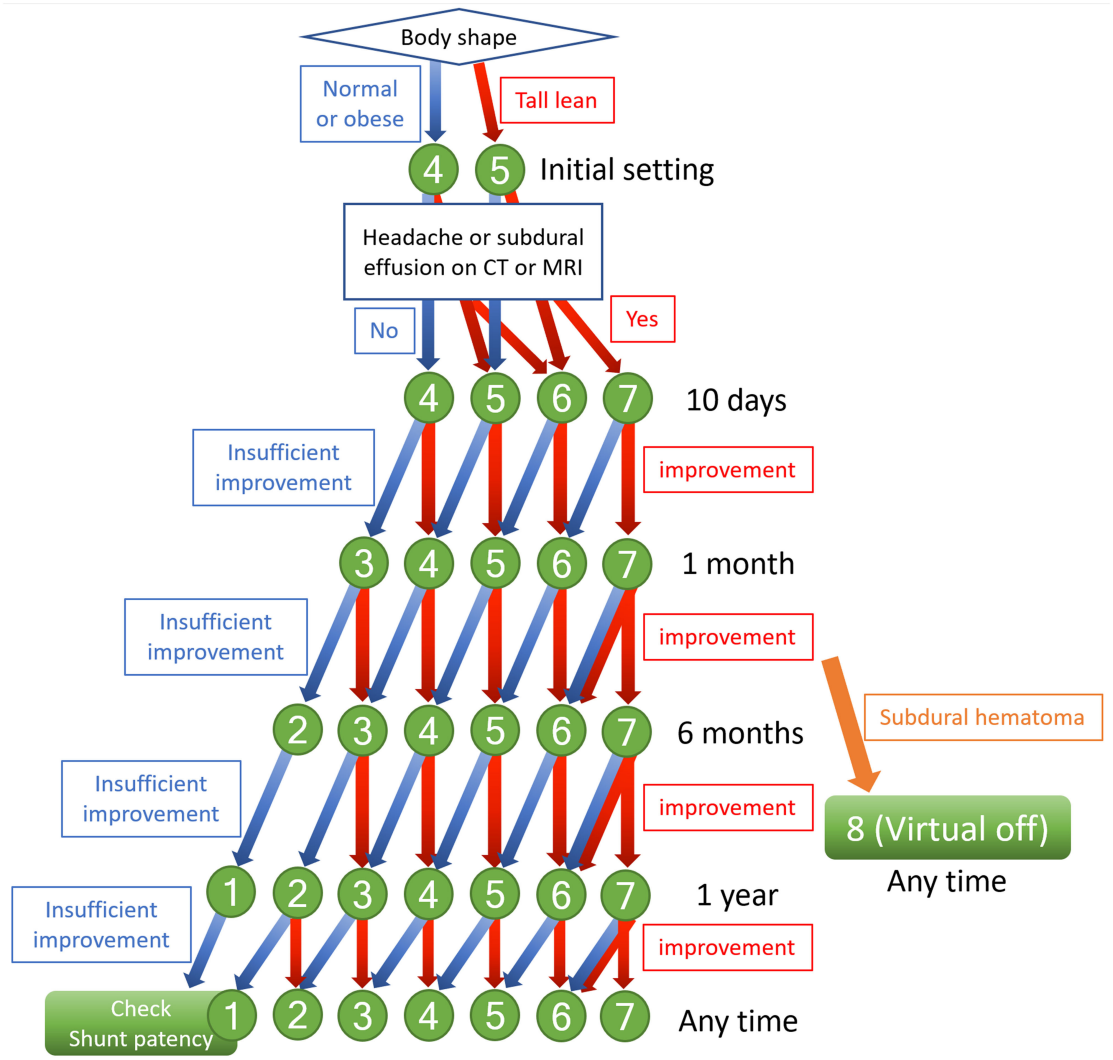
Flowchart of indicators to change shunt valve pressure. The numbers indicate the pressure setting of the CERTAS Plus Programmable Valve. Setting 1: 25 ± 20 mmH2O, setting 2: 50 ± 20 mmH2O, setting 3: 80 ± 20 mmH2O, setting 4: 110 ± 25 mmH2O, setting 5: 145 ± 35 mmH2O, setting 6: 180 ± 35 mmH2O, setting 7: 215 ± 35 mmH2O, and setting 8: above 400 mmH2O.

Since 2015, we have routinely used the CERTAS Plus Programmable Valve with Siphonguard Anti-Siphon Device in VP shunt surgery with our iNPH patients. The initial shunt valve pressure is set at medium pressure (4: 110 ± 25 mmH_2_O) for iNPH patients with normal body shape but optionally set one level higher (5: 145 ± 35 mmH_2_O) for tall lean patients. Within 10 days of VP shunting, the optimum valve pressure for each patient is determined by increasing one or two levels in those presenting with postural headaches or asymptomatic subdural effusion on a CT scan, as shown in flowchart of Figure 6. One month after VP shunting, the symptoms were not sufficiently improved or worsen, the valve pressure should be carefully decreased one level. We believe that this pressure setting protocol for the CERTAS Plus Valve can improve patients' symptoms early after shunt surgery, although we have not compared it with other protocols.

### Long-Term Shunt Valve Pressure Reduction After Initial Optimal Setting

Many studies have examined the optimal initial pressure setting of programmable-pressure shunt valves and adjustments early after shunting, but none have focused on long-term changes of the valve pressure more than half a year after shunting. In patients with severe symptoms, the remaining symptoms often worsen after several years following surgery, even when symptoms are initially improved by setting the optimal valve pressure early after shunting. In such patients, it is recommended that the valve pressure is reduced one level as the cause is likely to be under-drainage. However, it would be preferable if such patients could be identified in advance and the valve pressure lowered before the worsening of symptoms. Therefore, we postulate that the optimal pressure in the early period after shunting might not be the same as the optimal pressure in the medium to long-term. Compared to secondary NPH subsequent to subarachnoid hemorrhage (77), iNPH may have a longer period from CSF accumulation to the appearance of symptoms (45). It may take some time for normal CSF distribution and brain formation to return (54). Based on this evidence, if the expected improvement in symptoms and radiological findings are not observed 6 months after shunting, the shunt valve pressure should be reduced one level from the initial optimal valve pressure 6 months to 1 year after shunting (Figure 6). When the valve setting is reduced, patients should be checked for symptoms of over-drainage and subdural effusion or hematoma on a CT scan within 1 month. Using this aggressive long-term pressure-lowering strategy, we have experienced many patients who have improved beyond expectations and few adverse events such as re-over-drainage. We hope to confirm the effectiveness of these aggressive pressure-lowering approach in future clinical studies.

### Surgical Complication

The adverse events associated with VP shunt surgery in iNPH are listed as shunt malfunction (shunt pathway obstruction and mechanical failure), subdural hematomas, infection, cerebral hemorrhage around the ventricular puncture route, and symptoms of intracranial hypotension (e.g., headache, nausea, vomiting, dizziness, and tinnitus). In a systematic review of papers published before 2000, the mean complication rate of was 38% (range, 5–100%), and 22% (range, 0–47%) of patients required additional surgery (78). However, a systematic review of 28 studies published from 2006 to 2010 reported that the shunt revision rate was 13% and the rate of other surgical complications including subdural hematomas and infection was 8.2% (79). In the recent multicenter prospective cohort studies, the rate of serious adverse events had been reported to be 15–22% (9, 11), and 10–16% in the community-based retrospective studies (56, 80). Therefore, the complications of VP shunt surgery in iNPH have steadily decreased in recent years due to improvement in shunt technology and surgical techniques. However, the complication rate is still over 10%, which is too high for surgery aimed at improving the function of the elderly patients with an average age of 75 years. Since the surgical complications leads to the unfortunate outcomes that the symptoms do not improve as expected, neurosurgeons need to seriously improve the technique of VP shunt surgery to further reduce complications.

## Discussion

Delays in the diagnosis and treatment of progressive diseases are harmful to patients. The effects of CSF tap test or spinal drainage test for the iNPH diagnosis are transient, and symptoms return after a while (15). The effects of the CSF tap test has been reported to be associated with the duration since the onset of symptoms, and the effect diminishes as the disease duration increases (37). Therefore, even if the CSF tap test is repeated palliatively every time the symptom worsens, the same effect as the first one is not obtained, and there is a high possibility that the symptoms gradually progress and the timing of treatment is missed. For early diagnosis and early intervention, there is a need for widespread awareness that gait disturbance, cognitive impairment, and urinary incontinence or urgency is typical symptoms of iNPH in the elderly. Recently, Andrén et al. reported that delays in the surgical planning of shunting for iNPH patients create 2.57 times higher risk of mortality than early treatment (81). Crude mortality rates of 4 years occurred in 39.4% of patients waiting for shunt surgery for 6–24 months (mean: 13 months) and 10.1% in the patients treated within 3 months. In addition, iNPH patients are known to be at increased risk of falls due to imbalance and gait disturbance. The Idiopathic Normal Pressure Hydrocephalus Comorbidity and Risk Factors Associated with Hydrocephalus (iNPH-CRasH) study by the Swedish Hydrocephalus Quality Registry reported that the frequency of recurrent falls decreases from 67% before shunting to 35% after shunting, although this is still higher than the 11% risk in the general population (82). They also reported that iNPH patients feared falling more often than the general population even after shunting. The reduction of quality of life and independent living expectations due to fear of falling leads to frailty, social withdrawal, and impaired healthy life expectancy. Therefore, patients should be provided with additional interventions such as rehabilitation exercises and home safety care education after shunting. Especially, patients who had a complication of lower limb disuse syndrome and an impaired daily life due to moderate or severe gait disturbance are recommended actively perform rehabilitation even if the hospitalization period is extended, rather than being discharged as soon as possible after CSF shunting. It is expected that a better quality of life will continue even after discharge, if the patients are discharged after recovering until living their daily life without needing assistance at home and until the fear of falling disappears.

In conclusion, patients diagnosed with iNPH are referred to the hospital with the expectation that “dementia, gait disturbance, and urinary incontinence will be cured.” However, elderly iNPH patients with frailty syndrome, especially those with a history of stroke or comorbidity of Alzheimer's disease, may not get the therapeutic benefits they expect. Surgical intervention should be considered after fully explaining to the patient and their caregiver that the possibility of exacerbation is not unexpected. We should treat iNPH patients with the following points in mind: early detection, accurate diagnosis, consideration of surgical indications by a neurosurgeon experienced in iNPH treatment, the best treatment for each patient (including surgical method and adequate adjustment of shunt valve pressure), and post-shunting rehabilitation and long-term follow-up. We hope to work together in the future with specialists in the departments of neurosurgery, neurology, psychiatry, and rehabilitation to further develop and improve treatments for iNPH patients.

## Author Contributions

SY: conception and design. MI, MN, and KN: critical revision of the manuscript. All authors contributed to the article and approved the submitted version.

## Funding

This study received funding from Fujifilm Corporation for 4 years, beginning 2019; from the G-7 Scholarship Foundation in 2020; from Taiju Life Social Welfare Foundation in 2020; and the Japan Society for the Promotion of Science, KAKENHI for 3 years, beginning in 2021(Grant number: 21K09098). The funders were not involved in the study design, collection, analysis, interpretation of data, the writing of this article or the decision to submit it for publication.

## Conflict of Interest

SY has received research grants from the Japan Society for the Promotion of Science, KAKENHI for 3 years, beginning 2021; from Fujifilm Corporation for 4 years, beginning 2019; from the G-7 Scholarship Foundation in 2020; and from Taiju Life Social Welfare Foundation in 2020. He received a speaker's honoraria from Integra Japan, Fujifilm Medical Systems, Medtronic, Inc., and Nihon Medi-Physics Co., Ltd. MN received a speaker's honoraria from Integra Japan, Janssen Pharmaceutical K.K., and Medtronic, Inc. These were unrelated to the submitted work. KN has received grants from the Japan Agency for Medical Research and Development (AMED), a KAKENHI grant from the Japan Society for the Promotion of Science, and speaker's honoraria from Pfizer, Japan Inc., and Daiichi Sankyo Co., Ltd. These were unrelated to the submitted work. The remaining author declares that the research was conducted in the absence of any commercial or financial relationships that could be construed as a potential conflict of interest.

## Publisher's Note

All claims expressed in this article are solely those of the authors and do not necessarily represent those of their affiliated organizations, or those of the publisher, the editors and the reviewers. Any product that may be evaluated in this article, or claim that may be made by its manufacturer, is not guaranteed or endorsed by the publisher.

## Supplementary Material

Supplementary Video 1 Preoperative simulation.

Supplementary Video 2 Setting position.

Supplementary Video 3 Operation.
